# A quantitative study on the effects of an interactive multimodal application to promote students' learning motivation and comprehension in studying Tang poetry

**DOI:** 10.3389/fpsyg.2023.1189864

**Published:** 2023-06-08

**Authors:** Chuang Chen, Nurullizam Jamiat

**Affiliations:** ^1^School of Art and Design, Zhengzhou University of Industrial Technology, Zhengzhou, China; ^2^Centre for Instructional Technology and Multimedia, Universiti Sains Malaysia, Penang, Malaysia

**Keywords:** learning motivation, learning comprehension, interactive multimodal application, Tang poetry, interactive learning, educational technologies

## Abstract

Studying China's Tang poetry is a crucially integrated part of the language curriculum in primary schools because it is an important part of its cultural heritage and classical literature. However, due to the fact that Tang poetry is written in classical Chinese, which is quite different from modern Chinese Mandarin, and the complex categories of this poetry style, learning Tang poetry can be a challenging experience for many students. To address this problem, this study developed an interactive multimodal application based on the cognitive-affective theory of learning with media to learn Tang poetry in an interactive way. In order to assess the effectiveness of this method, a pretest-posttest control group experiment was conducted. The experiment included eighty third-grade students randomly and equally divided into experimental and control groups from an elementary school in Xinzheng, Henan Province, to test (1) whether the interactive multimodal application improves students' reading comprehension of Tang poetry and (2) whether the application enhances students' intrinsic and/or extrinsic motivation in learning Tang poetry. A multimodal interactive application was used by the experimental group to learn Tang poetry, while the control group used a traditional classroom method. According to the study's findings, it was found that students' intrinsic motivation and comprehension of Tang poetry improved through the use of the interactive multimodal application mode.

## 1. Introduction

Tang poetry, in Chinese classical literature, is a style of poem writing popularized mainly during the Tang Dynasty (618–907 AD), when it is often referred to as the “golden age” of Chinese poetry (Cheng, 2017). Besides being a valuable cultural heritage of the Chinese people, Tang poetry has long been an essential part of language instruction in Chinese primary and secondary schools (Wei and Geng, 2022). According to the language curriculum standards set by the Ministry of Education of the People's Republic of China, primary school students from grades 1 to 6 are required to have the ability to recite and understand 75 poems while developing the ability to feel, imagine, and understand the deeper meaning of these pieces (MOE of PRC, 2022). The study of Tang poetry thus becomes an essential part of the daily language curriculum and a compulsory learning objective for all students. In addition to being an effective tool for contemporary education, the study of Tang poetry has great cultural significance, allowing students to delve deeply into the culture and history of China's Tang Dynasty. This practice ensures the transmission of China's rich heritage and enhances students' character and patriotic sentiments while fostering their aesthetic awareness and proficiency in communication through the exploration of the language of Tang poetry (Chen and Lin, 2016).

However, students sometimes experience difficulty learning Tang poetry. The difficulty comes from the fact that these poetries are written in classical Chinese, which was used hundreds of years ago, and the language use of classical Chinese is quite different from Modern Chinese Mandarin. Also, the variation of forms in Tang poetry is another factor hindering students' comprehension. For example, Wuyan Jueju, a pentasyllabic quatrain containing only 20 words in its entirety, conveys complex and profound meaning with highly summarized Chinese words (Owen, 2018). Both of these could be challenges for students who are unfamiliar with Tang poetry when learning. In traditional classroom instruction, most teachers require students to learn Tang poetry through reading aloud and recitation (Chen and Lin, 2016). Additionally, most teachers fail to provide much background information about Tang poetry when teaching, for example, what they are written about, who they are written for, and why and how they are written. These all lead to difficulties in understanding its meaning. Therefore, students in lower grades may experience cognitive overload in such a traditional teaching method, which may have a negative impact on their interest in learning Tang poetry.

With the increasing popularization and advancement of digital technology and mobile devices, the domains of education have experienced dramatic changes, including innovative changes in language pedagogy. Multimodality, or a “multimodal interactive learning environment” (Moreno and Mayer, 2005, p. 5), is introduced to classroom teaching, where the images and text presented are determined by the learners' activities during the learning process. Compared to traditional learning environments, it has the ability to attract the attention of students, thereby engaging them in learning and ultimately achieving educational goals (Lina et al., 2019). Moreover, through verbal and non-verbal channels (e.g., using computers and projection equipment with a combination of text, pictures, sound, and animation), it enriches the content of teaching and thus stimulates learning (Hsiao and Lo, 2016). With the advent of touch-screen technology, interaction has become more direct and simple. Teachers can provide real-time feedback and guidance to students using an interactive multimodal application (hereafter, the IMA) with interactive features (Barnyak and McNelly, 2016). As opposed to explaining the learning process solely through verbal and non-verbal forms, the IMA allows students to participate, choose different learning strategies, and receive system feedback based on their actions (Cukurova et al., 2020).

IMA is an innovative digital teaching tool that integrates multimedia features and interactive functions to provide an enriched and engaging learning experience for students studying Tang poetry (Chen and Lin, 2016). This approach offers educators the ability to create a dynamic learning environment by incorporating text, pictures, sound, and animation to promote interactive, active learning and knowledge acquisition from multiple sources (Mayer, 2005). Unlike traditional multimedia instruction, which involves one-way knowledge transfer, IMA enables increased teacher-student interaction to take into account background knowledge, learning processes, knowledge testing, feedback, and reflection in an engaging and interactive way (Moreno, 2006b). This approach has been proven effective in enhancing students' learning outcomes and is particularly relevant for teaching Tang poetry (Moreno and Mayer, 2005; Moreno, 2006a).

The literature review shows that IMA has been used in teaching and learning activities (Chen and Lin, 2016). However, there are few studies on the use of these tools for learning Tang poetry, and various apps with the function of learning Tang poetry are mostly for entertainment, which may not be suitable for classroom use. Therefore, this study develops an IMA for learning Tang poetry based on a cognitive-affective model with audio, graphics, animation, fun tests, and practice. In addition, an experiment was conducted in an elementary school in Xinzheng, Henan Province, China to evaluate the effectiveness of the IMA.

## 2. Literature review

### 2.1. Tang poetry study and IMA

With advancements in technology, children now have the opportunity to access stories through various mediums such as television, computers, CDs, DVDs, and the internet (Takacs et al., 2015). More recently, the availability of apps on tablets or smartphones, such as the iPad or iPhone, has enabled access to these materials (Richter and Courage, 2017). Along with these advances, interactive e-books or interactive multimodal applications (IMA), specifically on iPads or other tablets, have become a crucial aspect of the learning ecosystem for children (Christ et al., 2019). Multiple empirical studies have shown that such advancements can positively impact students' motivation to read (Korat and Falk, 2019), reading comprehension (Bus et al., 2019, 2020), vocabulary acquisition (Kucirkova, 2019), and story retelling ability (Locher and Pfost, 2020). Additionally, researchers are now exploring the potential of IMA to enhance students' motivation and learning abilities in other subject areas.

Since Chinese poetry education mainly serves a pedagogical purpose, students frequently encounter challenges when attempting to appreciate poetry. As a solution, numerous researchers have suggested adopting educational technology methods to aid learners in comprehending poetry, such as implementing style recognition (Shih et al., 2012) and poetry comprehension (Yang et al., 2011). Sun and Cheng's (2007) study shows that using rich media in e-learning courses for Chinese poetry resulted in significantly better learning outcomes than courses with less rich media. Therefore, providing students with reliable and easily accessible multimedia resources containing highly rich media is essential to improving their learning experience (Sun and Cheng, 2007). Chen and Lin (2016) created a digital game-based multimodal interactive application for Chinese poetry education. The resulting findings revealed that students who utilized the game-based interactive multimodal application outperformed those who relied on conventional classroom teaching methods (Chen and Lin, 2016). Another study conducted by Yang et al. (2013) found that the use of multimedia resources for contextualized Tang poetry learning had significant benefits. The majority of students expressed satisfaction with the proposed system, and the teacher participant displayed a favorable attitude toward utilizing this system (Yang et al., 2013).

Alongside IMA, various other technologies, such as virtual reality and digital games, are also being explored for integration with educational content (Chuang and Chen, 2009). It is worth noting, however, that incorporating augmented reality, virtual reality, and digital game content can be a challenging task for language teachers who lack programming skills. This includes difficulties in terms of both teaching preparation time and activity development (Jdaitawi and Kan'an, 2022). Some researchers have highlighted the expense, complexity, and specialist expertise required to create content in these digital technologies, which ultimately adds to the workload of teachers and students (Folkestad and O'shea, 2011; Akçayır and Akçayır, 2017). Compared to these, IMA is a simpler, less expensive, and easier-to-use teaching and learning technology. Many design platforms now offer abundant resources for creating IMAs that are specifically tailored to their content (Chen and Lin, 2016). For example, Yoya is an IMA development software designed by Xiamen Elegant Web Technology Co. that enables teachers to create IMAs for teaching and learning content directly from existing resources.

### 2.2. Motivation and comprehension in learning

Motivating students to learn is one of the key principles of efficient education (Kim and Frick, 2011). Motivation to learn indicates a students' desire to participate in and learn from training activities (Meece et al., 2006).

The effect of motivation on learning has always been a central issue in educational psychology. Ryan and Deci (2000) categorizes motivation into two dimensions: intrinsic motivation and extrinsic motivation. As defined by motivation theory, intrinsic motivation for learning is the desire to learn for the satisfaction or benefit it provides. Students who have a higher level of intrinsic motivation are more likely to persist in engaging with complex problems and to gain knowledge from their slips and mistakes (Jovanovic and Matejevic, 2014). Extrinsic motivation, on the other hand, refers to reasons that are not directly related to learning activities or knowledge content, for instance receiving praise, receiving a good scores, or outperforming others (Schiefele et al., 2012).

Learning motivation is essential to the development of learners' literacy skills (Chuang and Jamiat, 2023). Learners' motivation has a greater influence on the outcomes of learning activities than cognitive characteristics, intelligence, and prior knowledge (Bates et al., 2016). Having a positive sense of control over their learning activities enhances their intrinsic motivation. In this sense, students are more likely to persist in difficult academic tasks, so there is a possibility for them to have deep processing of newly-presented knowledge (Cartwright et al., 2016). Thus, the possibility of knowledge retention also increases.

Motivation also has positive effects on students' comprehension of reading texts. It has been demonstrated that motivated students read more and are willing to spend more time reading than their less motivated peers (Kpolovie et al., 2014). Several researchers have argued that motivation plays a significant role in reading comprehension and that learners' motivation can have a significant influence on their reading behavior and ability (Ghalebandi and Noorhidawati, 2019; Wong and Neuman, 2019). Such effects can also be manifested through students' reading scores. In a study performed by Bus et al. (2019), it was shown that the learning motivation of students can significantly affect their reading comprehension scores. Similarly, promoted motivation may also positively influence students' reading fluency and reading skills (Zhou and Yadav, 2017). However, it should be noted that the popularization of technology also changes the research focus from the relation between motivation and reading comprehension in printed materials to e-book reading. Electronic devices greatly change our daily lives, and reading for learning is no exception. In an empirical study, Korat (2010) analyzed the reading motivation and comprehension scores of student participants who used paper books and e-books. Based on the results of the study, students using e-books had higher self-reported motivation scores than students using paper books (Korat, 2010). Furthermore, e-book-preferred students with higher reading motivation scores outperformed those using paper books in terms of reading comprehension since e-books are interactive and offer a variety of multimedia features, so students can interact with them and learn at their own pace (Richter and Courage, 2017).

### 2.3. Interactive multimodal learning environments

A multimodal learning environment combines verbal and non-verbal modes of knowledge presentation, through which learners receive both verbal and visual content representations (Paivio, 1990). It has been demonstrated that in such an environment, students' understanding of knowledge can be improved when compared with that in conventional teaching and learning (Fletcher and Tobias, 2005). It is also considered to be more effective in combining verbal and non-verbal modes when compared to either of the modes in presenting knowledge individually (Moreno, 2006b), which could ease the load on students' cognitive processing (Sweller, 2005). In other words, verbal and non-verbal modes are not contradictory; they, however, are supplementary. Therefore, the application designed in this study combines verbal, non-verbal, and picture representations in parallel, for example, when a poetry is being read automatically and its corresponding caption is displayed on the screen at the same time.

When it terms to multimodal interactive environments, the distinguishing characteristic of this type of learning environment is its interactivity, compared with multimodal environments. Multimodal learning environments present multimedia content in a predetermined way, and learners passively receive information without interacting with it (Markus, 1987). There are multiple examples of non-interactive multimodal learning environments, including the use of simulated animations by teachers or the use of courseware containing illustrations and text (Wagner, 1994). However, when learning occurs in a multimodal interactive environment, the process of learning is determined by the learner's actions (Moreno and Mayer, 2007). In other words, the pace and rhythm of learning are controlled by the learner himself or herself. The interactivity function plays a vital role in the Interactivity Multimodal Application (IMA), and it is responsive to the students' activities during the study process (Moreno and Mayer, 2007). According to previous research, interactive elements, such as hotspots and games, especially those unrelated to the learning content, can distract students' attention and then lead to poor performance in reading (Takacs et al., 2015; Bus et al., 2019; Danaei et al., 2020). However, some empirical studies have demonstrated that, as long as information is processed both visually and verbally, learning can be enhanced if the interactive elements are consistent with the content (Korat, 2010; Sun et al., 2019). For this reason, the IMA developed for this study includes a table of contents, a page turn function, feedback, and hotspots related to the learning content.

### 2.4. The cognitive-affective theory of learning with media

**Figure 1:**
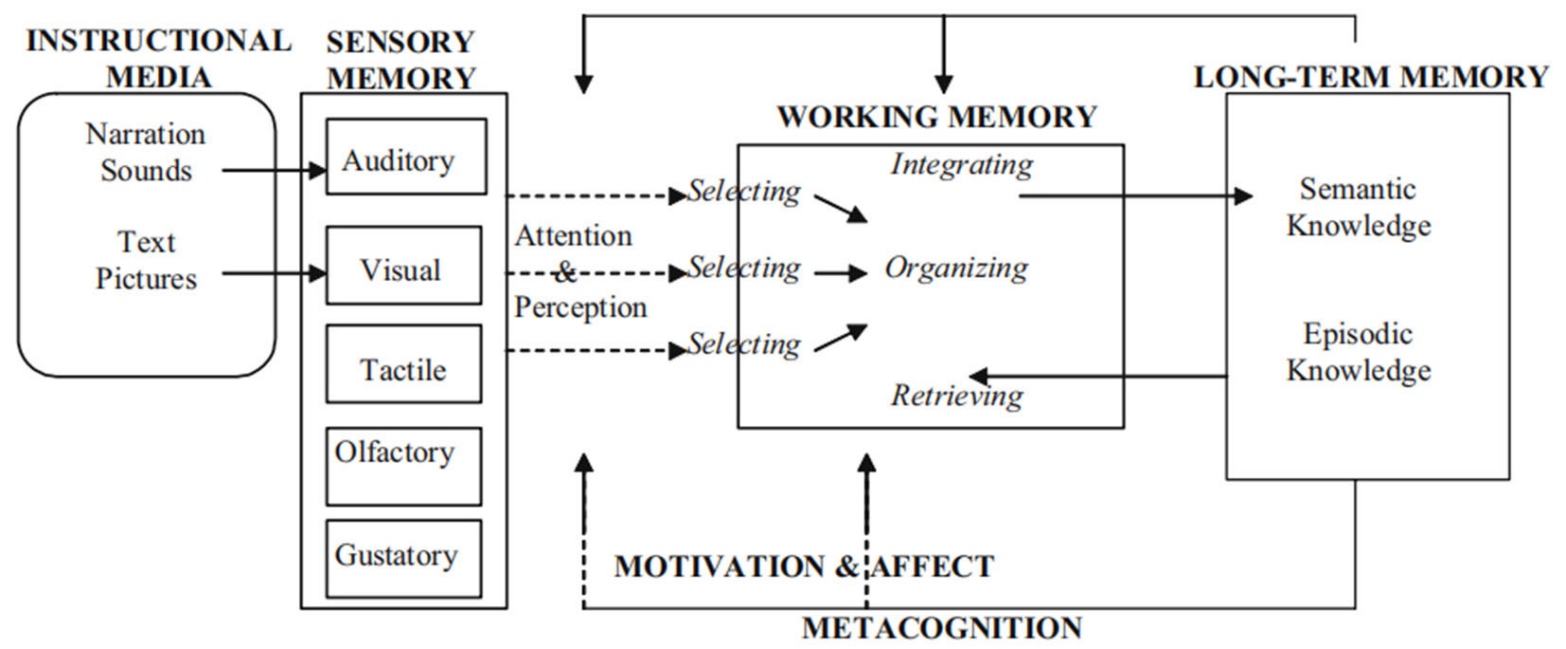
A cognitive–affective model of learning with media (Moreno and Mayer, 2005).

The cognitive-affective theory of learning with media (CATLM) extends the cognitive theory of multimedia learning (CTML) to VR, virtual agents, and situation-based interactive learning environments, which can provide learners with instructional materials and learning experiences that go beyond text and images (Moreno, 2005). Models and theories that integrate cognitive and affective processes are demonstrating a shift from the cognitive paradigm to the affective paradigm in multimedia learning and instructional design (Moreno, 2006a). Based on cognitive and motivational research, the CATLM makes the following assumptions: (a) learners process information through multiple channels (Baddeley, 1992); (b) working memory is limited and only a limited amount of information can be processed at any given time (Sweller, 1999); (c) meaningful learning occurs when learners select, organize, and integrate knowledge with their prior learning (Mayer, 2005); (d) the structure of long-term memory is dynamic and evolutionary (Tulving, 1977); (e) motivational factors play an important role in learning (Pintrich, 2003); (f) learning is regulated by metacognitive factors by modulating cognition and affect; and (g) when learners use media for learning, their prior knowledge affects their effectiveness (Moreno, 2004). Based on CATLM, Figure 1 illustrates a model for learning in an interactive multimodal environment.

As shown in Figure 1, instructional media may include both verbal and textual explanations as well as non-verbal modes of knowledge presentation, such as pictures and sounds. To further process working memory, students must first pay attention to and select relevant verbal and non-verbal information. Students then need to organize the information into a coherent mental model and integrate this organized information with their prior knowledge (Baddeley, 1992). Using an interactive learning method, learners' cognitive activities are guided partly by their activated prior knowledge and partly by the feedback and strategies for instruction embedded in the learning environment (Moreno and Mayer, 2007). In summary, CATLM offers a theoretical framework that focuses on recognizing how learning takes place through the use of instructional materials that go beyond just text and pictures (e.g., interactivity).

## 3. An IMA for Tang poetry learning

### 3.1. CATLM-based instructional design principles

Multimedia cognitive learning theory proposes cognitive principles of instructional design such as the principles of modality and verbal redundancy, personalization, temporal and spatial contiguity, coherence, and redundancy (Sorden, 2012). These principles have proven to be effective when applied to interactive multimodal learning environments. However, learners can interact with the instructional system through interactive multimodal environments, so principles of CATLM-based instructional design are also proposed (Moreno, 2006a). Additionally, corresponding design strategies are proposed for these educational design principles in the design of the IMA, as shown in Table 1.

**Table 1:** CATLM-based instructional design principles.

**Principle**	**Description**	**Strategies**
Pre-training	It is more effective for students to learn when they receive targeted pre-training that provides or activates relevant prior knowledge	Provide background knowledge about Tang poetry and poets in the teaching application
Pacing	It is more effective for students to learn if they are allowed to determine the pace at which instructional materials are presented	Provide navigation within the application as well as feedback and hotspots related to the learning content
Guided activity	A student learns more effectively when he or she is able to interact with instructional agents who support and guide the cognitive processing of the student	Design a virtual animated character to act as a teacher
Feedback	It is more effective for students to learn from interpretive feedback rather than from purely corrective feedback	Provide interpretive feedback and receive encouragement after students have chosen the correct answer
Reflection	A student learns better when he or she is asked to reflect on the correct answer as part of the process of creating meaning	Provide students with open-ended questions in the application to facilitate reflection

**Figure 2:**
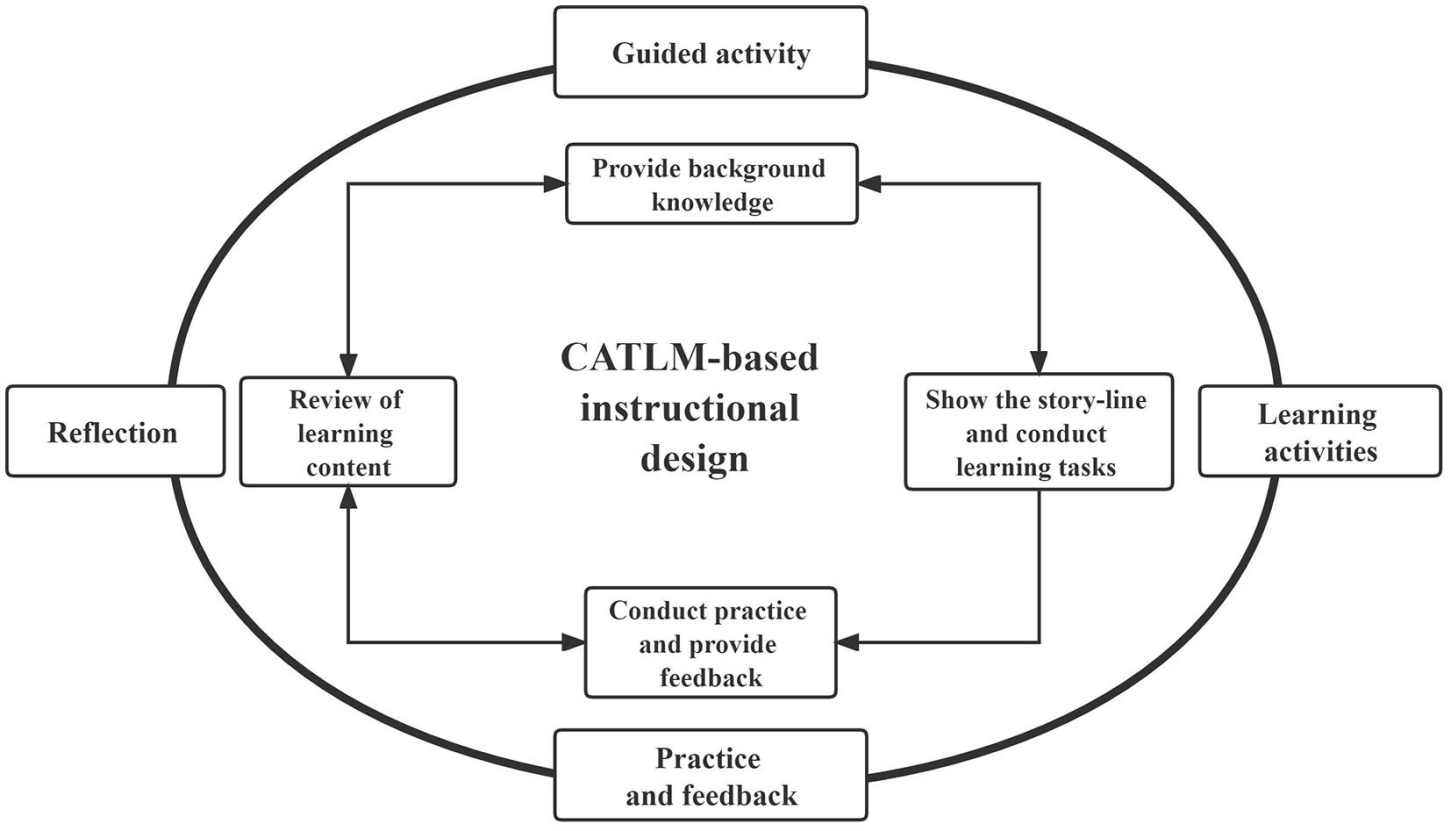
Model for IMA development based on the CATLM-based instructional design.

In this study, a model based on CATLM was used to develop an IMA for Tang poetry learning, an important part of the Chinese primary and secondary school curriculum for decades. According to Huang et al. (2022), the CATLM theory identifies the mechanisms that facilitate meaningful learning in multimodal learning settings when learners take direct action with instructional systems (e.g., dialogue, control, and manipulation). CATLM combines the strengths of various kinds of sensory interaction (e.g., auditory, visual, and tactile) with different aspects of individual function (e.g., cognitive, motivational, and affective) (Moreno and Mayer, 2005). Therefore, this study proposes a model for IMA development based on the CATLM principles of instructional design, as shown in Figure 2. The model contains four stages: guided activities, learning activities, practice and feedback, and reflection. The IMA design corresponding to these four stages includes providing background knowledge; showing the storyline and conducting learning tasks; conducting practice and providing feedback; and reviewing the learning content, respectively. There are two-way arrows connecting the four stages since the IMA's interactive function enables learners to switch between the stages at any time.

### 3.2. IMA design and procedures

**Figure 3:**
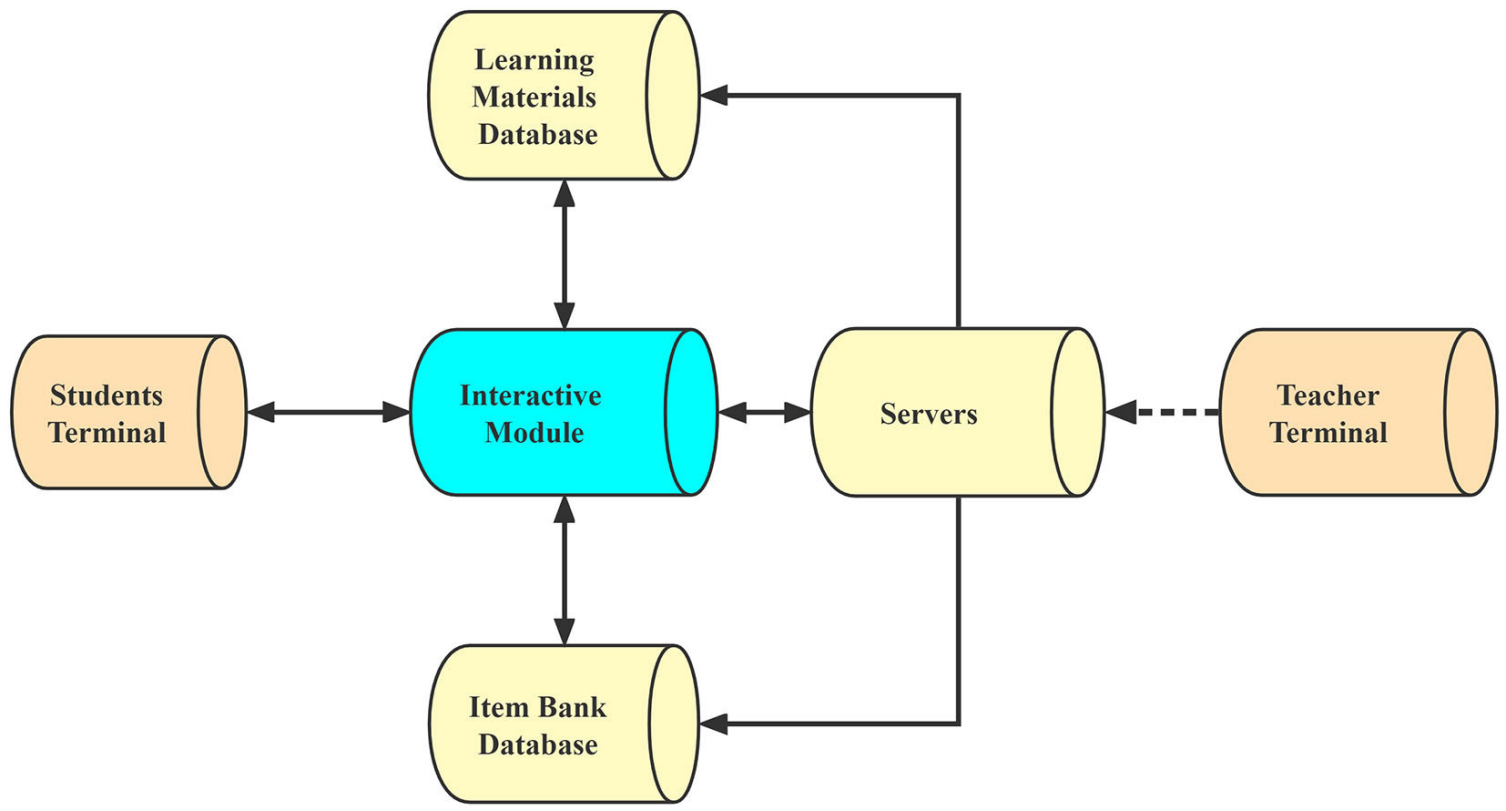
System structure.

This study used Yoya, an IMA development software developed by Xiamen Elegant Web Technology Co., to provide an interactive multimodal learning environment for students. Figure 3 shows the system structure. It consists of a teacher terminal, multiple student terminals, a database of learning materials, a database of project libraries, a server, and a module that allows interaction. The interactive module is the core of the IMA. It performs various interactive functions by responding to commands from the student terminals and accessing the learning materials database and the project library database through the server.

However, it should be noted that the teacher cannot modify the IMA content if it has been uploaded to the system through the teacher's terminal and transferred to those of the students. All materials are stored on Yoya's server. Interactive functions include presenting (1) knowledge using virtual character narration, animation, text, and images; (2) responding to commands from students (e.g., switching to another page; activating hotspots); (3) preparing exercises including multiple choices, “true or false”, sequencing, and fill-in-the-blank; and (4) reviewing (Ertem, 2011). All these interactive functions will be discussed and illustrated with details later in this section, attached to a case of selected Tang poetry.

**Figure 4:**
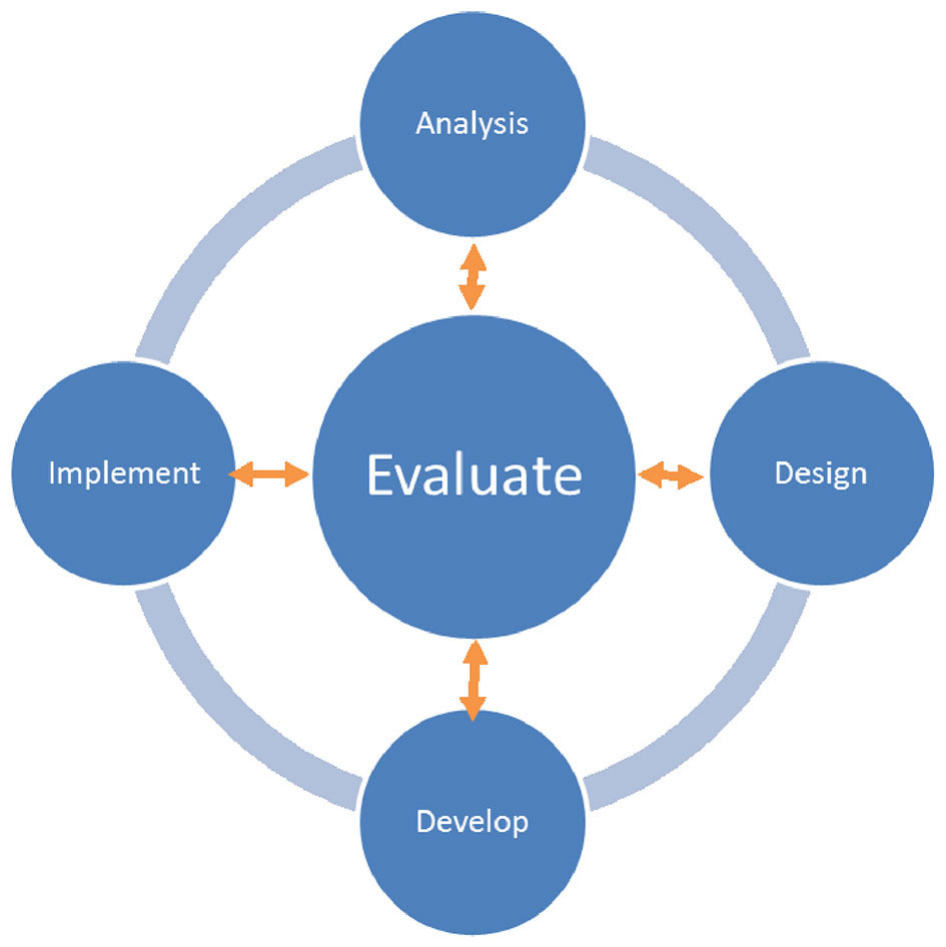
The ADDIE instructional design model (Branson, 1978).

**Figure 5:**
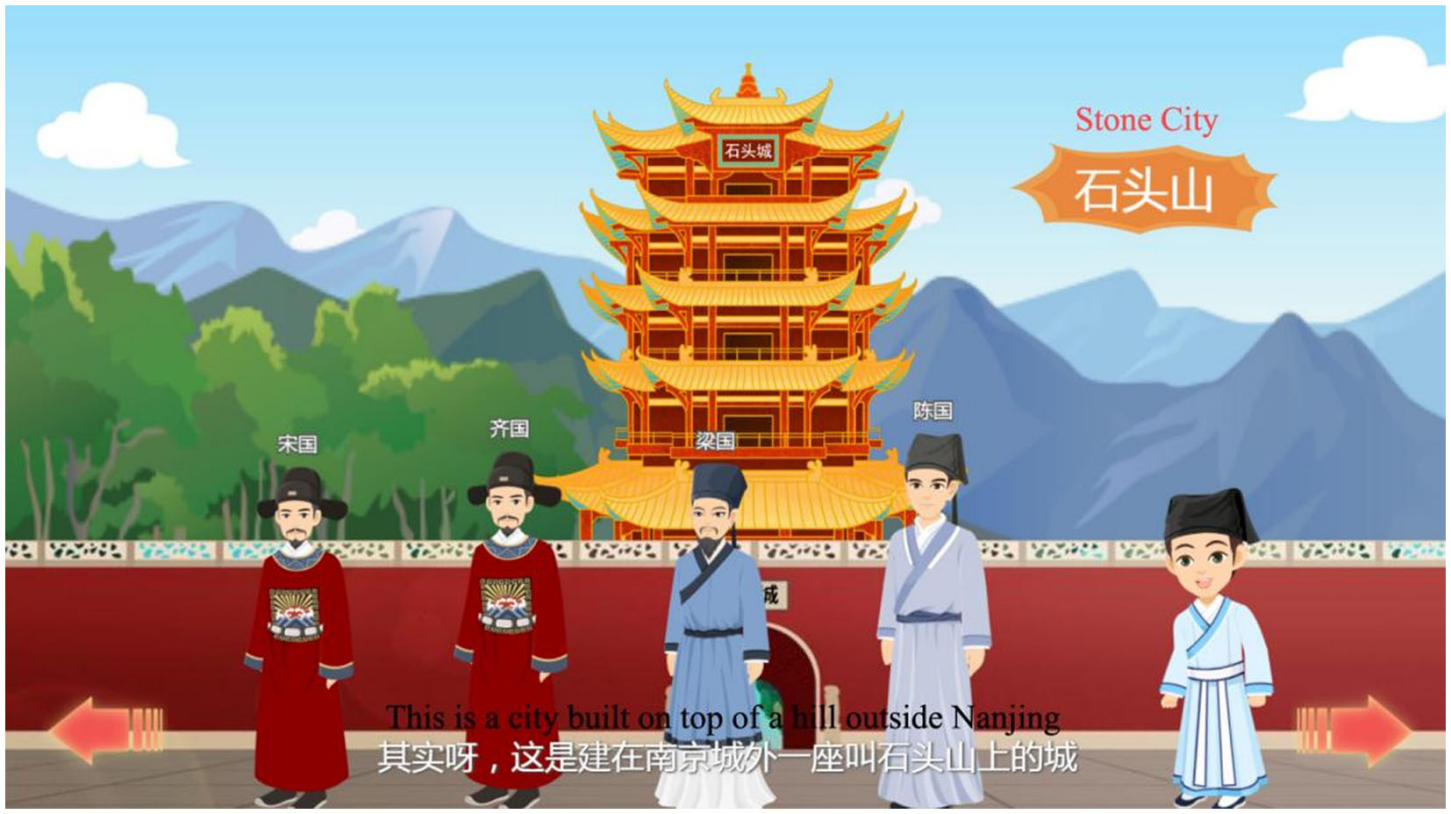
IMA interface, background story of the Tang poetry.

In this study, a famous poetry written by the Tang Dynasty poet Liu Yuxi, “The Stone City”, is selected as the learning material. The Analysis-Design-Develop-Implement-Evaluation (ADDIE) instructional design model (Branson, 1978) was adopted in the design and development of the IMA, as shown in Figure 4. This model represents the continuous cycle of phases associated with analysis, design, development, implementation, and evaluation. In each stage, there is an opportunity for iteration and revision before moving on to the next. Consequently, the IMA can be effectively analyzed and controlled, with its quality improved. To simulate the context of Tang poetry, the IMA adopted a storytelling format in its presentation after much discussion and literature reading. This would provide background knowledge about the selected Tang poetry (e.g., what this poetry is about, why it is written, and who it is written for), which may not be adequately managed in traditional teaching methods. Such inadequacy of background knowledge may result in a confusing learning experience (Moreno and Mayer, 2005), and thus it deserves attention. Moreover, this study developed a virtual animation character for the purpose of illustrating Tang poetry. Figure 5 shows the animated characters in this IMA.

#### 3.2.1. Provide background knowledge

At the beginning of the learning activity, the animated characters introduce themselves and guide the students through the learning. To facilitate the learning of the Tang poetry and assist students' comprehension on selected poetry, students are introduced to the history of Chinese dynasties, the historical background of the poet, and the context in which the poetry “Stone City” was written. As shown in Figure 5.

#### 3.2.2. Show the story-line and conduct learning tasks

**Figure 6:**
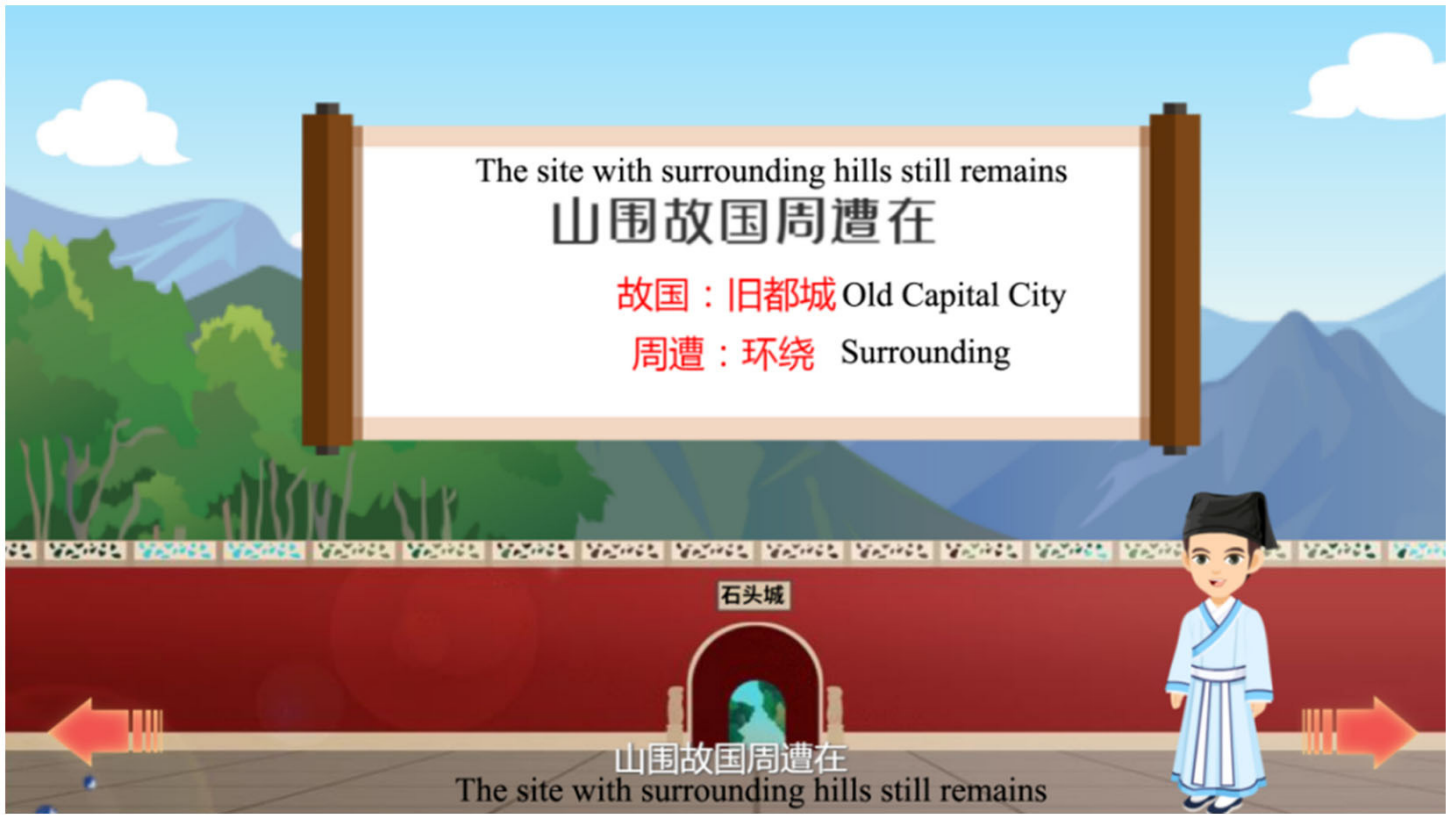
IMA interface, the content and interpretation of the Tang poetry.

The content and interpretation of the poetry are presented to learners after demonstrating background knowledge, which enables them to grasp the content and meaning of the poetry quickly. Learners can repeat a particular verse or line at any time by using the page-turn function. The core vocabulary is always explained in red, as indicated in Figure 6.

#### 3.2.3. Conduct practice and providing feedback

**Figure 7:**
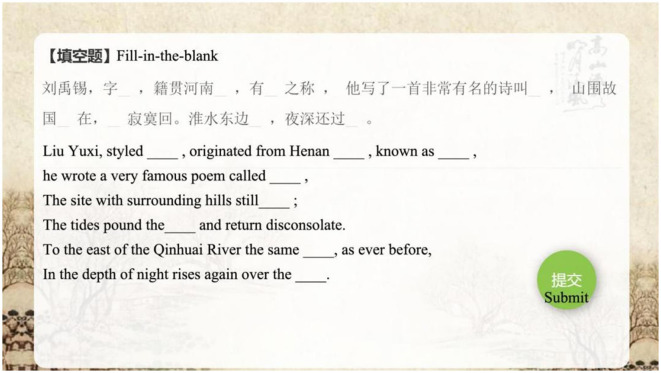
IMA interface, the practice of fill-in-the-blank tests.

The practice and feedback phase of the IMA involves competition, mainly offering fun tests. Students who successfully answer all questions receive an honorary title as a reward. In this way, learners remain motivated to learn, because interesting and competitive elements could foster motivation in learning. Moreover, according to Garris et al. (2002), providing feedback can guide students to concentrate on the learning and work hard to achieve their main goals with encouragement. After each question, students will receive their feedback corresponding to their answer. An example of a fill-in-the-blank question is given in Figure 7.

#### 3.2.4. Reviewing the learning content

**Figure 8:**
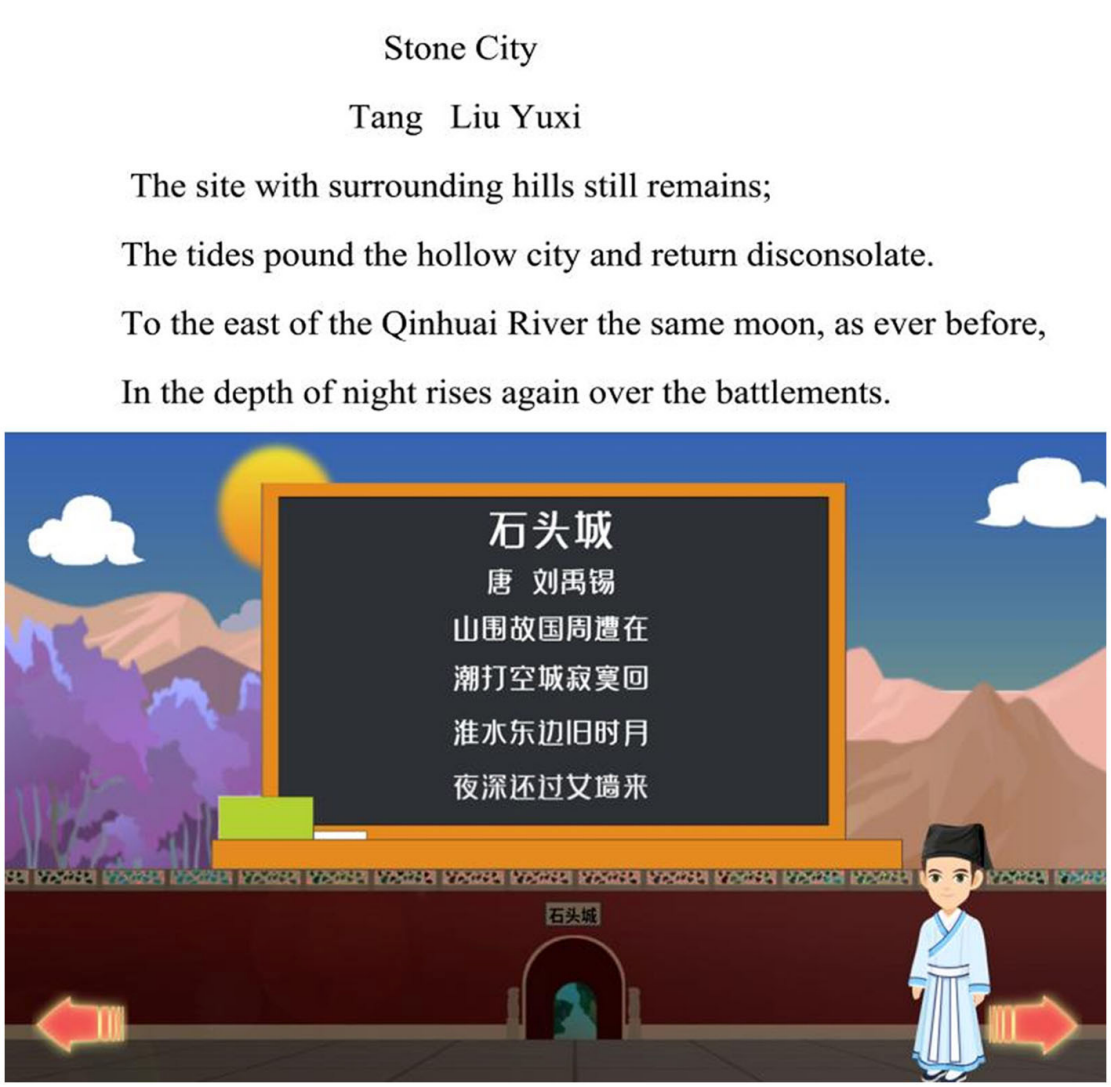
IMA interface, reviewing the learning content.

During the stage of reviewing, a summary is provided on the learning content in this session. Apart from the meaning of the poetry itself, learners should also be able to appreciate the meaning of the poetry, such as its social significance. Moreover, reflection on learning is also an integral part in this stage. According to Wigfield (1997), e-learning provides more opportunities for reflection, which helps people identify problems and find solutions. Through the process of reflection on learning content, for example, asking/encouraging students to reflect what they have learned and what is the connection between such learning and their daily life, learners are motivated to learn further, thereby fostering their interest in the content and improving learning outcomes. Assisted by the functions of content catalog and page skipping, learners can review any content they have learned previously at any time, which can contribute to enhancement of students' comprehension on Tang Dynasty poetries, as shown in Figure 8.

## 4. Research hypotheses

In this study, an IMA was designed to access the efficacy of the IMA on the students' motivation and comprehension when learning Tang poetry. The following are the research hypotheses for this study:

Hypothesis 1: Students in the experimental group were more motivated to learn than those in the control group.

Hypothesis 2: Students in the experimental group were more motivated to learn than those in the control group, both in terms of extrinsic and intrinsic motivation.

Hypothesis 3: Students in the experimental group had better comprehension than those in the control group.

Hypothesis 4: Students in the experimental group had better comprehension than those in the control group, both in terms of inferential and literal comprehension dimensions.

## 5. Materials and methods

### 5.1. Research design

**Figure 9:**
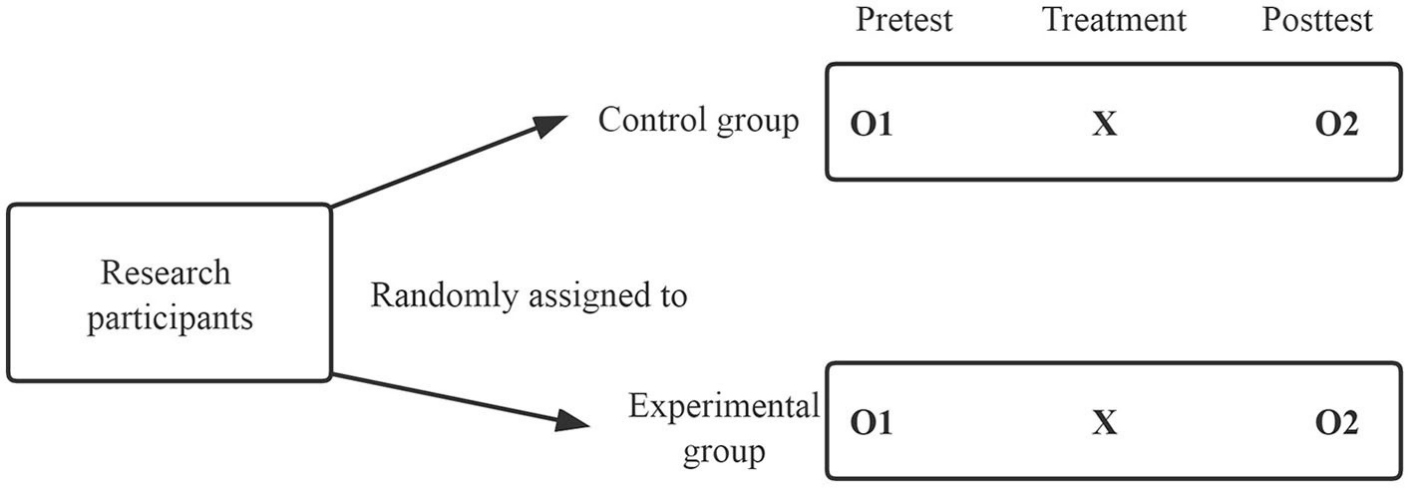
Pretest-posttest control group experiment design.

A pretest-posttest control group experiment was used in this study, in which participants were randomly assigned to the experimental (IMA methods for Tang poetry learning) and control (traditional classroom learning methods) groups (Valente and MacKinnon, 2017). As shown in Figure 9, the dependent variable O was first pre-tested (O1) for both groups, then the independent variable X was imposed, and finally the dependent variable was post-tested (O2) for both the experimental and control groups (Johnson and Christensen, 2019). An ANCOVA was used to identify whether the independent variable X would produce different results for the dependent variable O. This study used students' pre-test scores (O1) as a covariant. The pretest-posttest control group experiment is a well-designed experiment because it controls for competing assumptions about the internal validity of the threat experiment (Johnson and Christensen, 2019). The random assignment of participants maximizes the assurance that the groups are equal at the outset of the experiment.

### 5.2. Participants

A total of eighty third-grade students from a primary school in Xinzheng, Henan Province, participated in this study. They were selected using a one-stage cluster sampling technique, and eventually two classes out of five were selected. All participants share a language teacher and have had access to the school's computer courses since the beginning of their first year. In addition, each student has the necessary skills to use a tablet with ease. The experimental and control groups were randomly assigned. After random assignment to the control and experimental groups, the 80 students were coded as students 1 to 80 to ensure anonymity. Specifically, students 1–40 were in the control group, and the remaining forty, students 41–80, were in the experimental group. The basic concepts of Tang poetry were tested on both groups to ensure that they had the same prior knowledge.

### 5.3. Instruments

The Tang Poetry Comprehension Test (TPCT) was developed based on a curriculum guide attached to the textbooks of the People's Education Press (2018), which are commonly used in Chinese elementary schools. In addition, third-grade language teachers were invited to review and revise its content. The main intention of the pre-test was to assess the participants' basic knowledge of Tang poetry. A total of 50 points were awarded for five multiple-choice questions to test literal comprehension and five reading comprehension questions to test inferential comprehension ability. Each multiple-choice question is rewarded with 4 points, while 6 points are given for each reading comprehension question. The post-test was developed in the same way as the pre-test, with only different questions included. The TPCT includes knowledge about the poetry, the context in which the poetry was written, an interpretation of the verses, and a description of the poetry. In this sense, students are required to provide correct answers based on their comprehension of the content of poetry. The KR20 (Kuder-Richardson 20) of the Tang Poetry Comprehension Test (TPCT) is 0.79.

A questionnaire adapted from the Electronic Storybook Motivation Scale by Kao et al. (2016) is used in this study for students' learning motivation assessment. The original scale comprises 30 questions. The overall questionnaire had a Cronbach's alpha of 0.88, while the Cronbach's alphas for each sub-dimension were 0.82–0.93 (Kao et al., 2016). These indicate the reliability of the Electronic Storybook Motivation Scale. To meet the aim of assessing students' learning motivation in our study, two dimensions were selected: intrinsic motivation and extrinsic motivation. A five-point Likert scale was used in the questionnaire. Depending on their response, students selected either “strongly agree,” “agree,” “neutral,” “disagree,” or “strongly disagree,” with corresponding scores ranging from 5 to 1. A higher score is associated with greater motivation to learn, and vice versa. The Cronbach's alpha value of the electronic storybook motivation scale used in this study is 0.85.

The intrinsic motivation dimension consists of 8 items, for example, Item 1: “I find Tang poetry interesting from the beginning, so I am drawn to it”; Item 3: “I feel satisfied after learning Tang poetry because I gain a great deal of knowledge”; Item 5: “Tang poetry increases my knowledge, and I enjoy this sense of achievement”. For that on extrinsic motivation dimension, 7 items are included, for example, Item 9: “Whether I like Tang poetry or not, I don't want to upset my teacher by not studying well”; Item 11: “Studying Tang poetry makes me gain knowledge and can make my parents proud of me”; Item 13: “I want to be the best student in my class at learning Tang poetry so that I can surpass other students.” The score ranges from 15 as minimum (1^*^15 = 15) to 75 as maximum (5^*^15 = 75).

### 5.4. Experimental procedure

**Figure 10:**
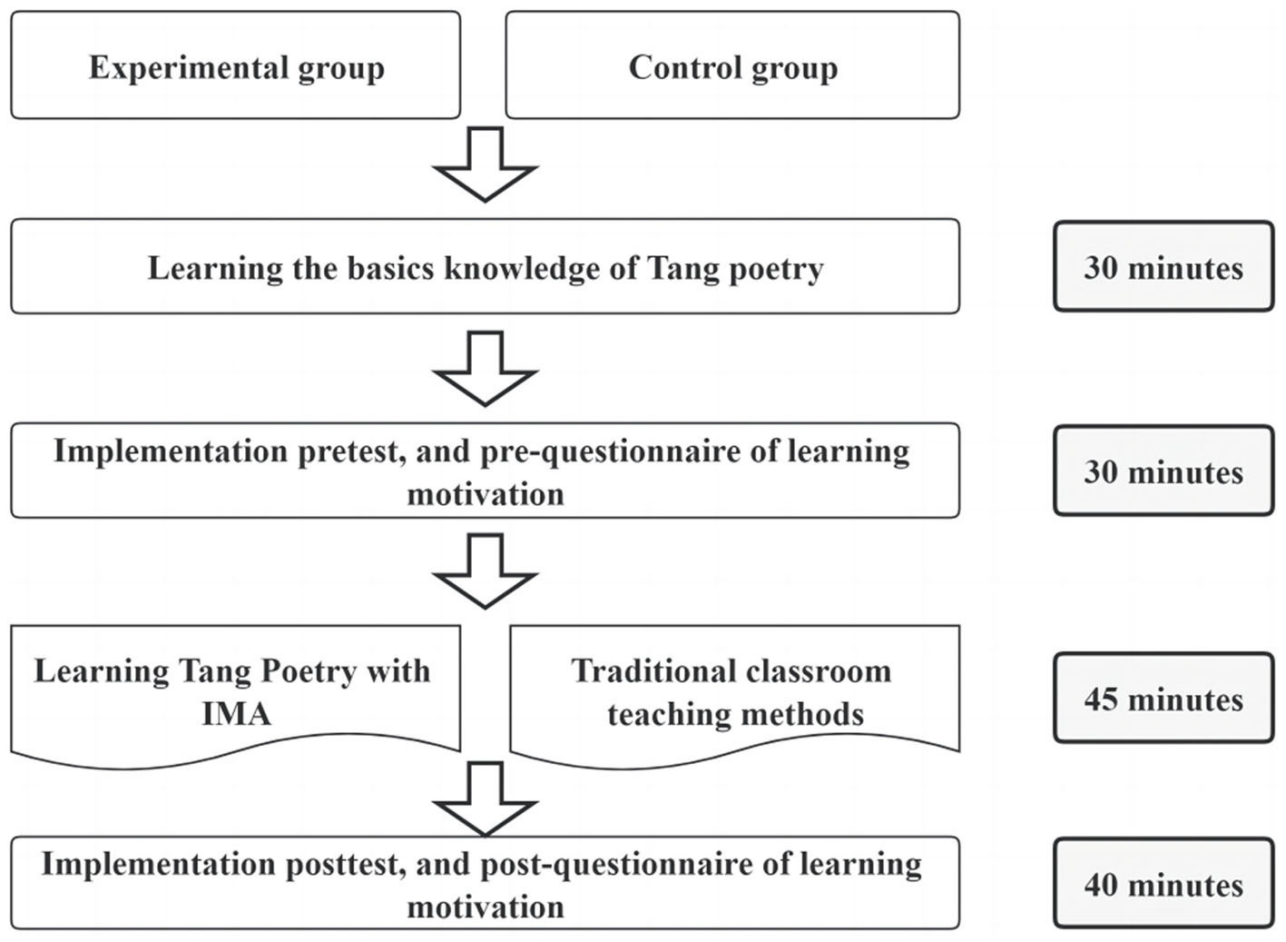
Experiment procedure.

The purpose of this study is to access the effects of an IMA on the students' motivation and comprehension when learning Tang poetry. Figure 10 shows the experiment's procedure. The study was conducted in a language classroom at an elementary school in Xinzheng, Henan Province, China. Prior to learning Tang poetry, students in both control and experiment groups were instructed in Tang poetry knowledge, including its classification, style, and grammar. This phase lasted for about 30 min. Following this, the pre-test and pre-questionnaire were administered to both groups of students. Students were required to finish the pre-test and pre-questionnaire within 30 min. Then, Tang poetry learning activities were conducted. Students in both groups studied the poetry “Stone City” by Liu Yuxi, with the control group using traditional teaching methods (PPT presentation, multimedia materials, following-up questions on reading comprehension and repeated reading) and the experiment group for IMA. The whole learning activities lasted for 45 min, similar to a lesson in Chinese elementary school. Following the learning activities, a Tang poetry post-test was given, as well as a post-questionnaire to assess learning motivation.

## 6. Data analysis and results

This study uses analysis of covariance (ANCOVA) to investigate the effects of IMA on the motivation and comprehension of students learning Tang poetry. One potential advantage of ANCOVA is that it allows for the control of extraneous variables that may be related to the dependent variable but are not of interest in the research question (Verona and Miller, 2014). By including these variables as covariants, ANCOVA can increase the statistical power and precision of the analysis by reducing the variance associated with the covariant, thereby increasing the sensitivity to detect the effects of the independent variable(s) on the dependent variable (Cochran, 1957). ANCOVA can also help improve the accuracy and validity of the estimates of treatment effects by reducing error variance and increasing the precision of the estimates (Jöreskog, 1988). In this study, ANCOVA uses students' pre-test scores as a covariant to control for the influence of pre-test scores on post-test scores. As a result, the experimental results obtained are more accurate. All hypotheses formulated for this study were tested at a significance level of 0.05. In the analysis, a *p* ≤ 0.05 indicates that the hypothesis was accepted. Otherwise, the hypothesis is rejected (*p* > 0.05).

In order to apply ANCOVA, the following assumptions must be fulfilled: (a) Normality: The dependent variable must be continuous and normally distributed within each group. (b) Homogeneity of variances: The variance of the dependent variable should be equal across all groups of the independent variable. (c) Linearity: The relationship between the dependent variable and the covariate should be linear. (d) Independence: Observations within each group must be independent of each other and the covariate. (e) Homogeneity of regression slopes: The slopes of the regression lines should be equal across groups (Stevens, 2013).

### 6.1. Learning motivation

In this section, we measure whether there is a significant difference in motivation between the experimental group using the IMA for Tang poetry learning and the control group using traditional classroom teaching methods. The study also examined the intrinsic and extrinsic motivation of students in both groups. The post-test scores were analyzed using ANCOVA using the pre-test scores as covariant.

The homogeneity of regression slopes test and homogeneity of variance were performed on the motivation scores of the two groups to meet the basic assumptions of the analysis of covariance (ANCOVA). According to the results, the assumptions of homogeneity of regression slopes (F = 0.27, p = 0.608 > 0.05) and Levene's test of equality of error variances (p = 0.26 > 0.05) were satisfied and could be analyzed for ANCOVA. In addition, intrinsic and extrinsic motivation scores were tested for homogeneity of regression slopes and homogeneity of variance. The test results showed that the assumption of homogeneity of regression slopes was not significant with intrinsic motivation (F = 0.26, *p* = 0.61 > 0.05) and extrinsic motivation (F = 0.33, *p* = 0.57 > 0.05). At the same time, the results of Levene's test of equality of error variances were satisfied with intrinsic motivation (*p* = 0.87 > 0.05) and extrinsic motivation (*p* = 0.38> 0.05). This indicates that these two dimensions can be analyzed using ANCOVA.

An analysis of the ANCOVA results for two groups of students is presented in Table 2. There was a significant difference in post-test motivation scores between the experimental and control groups [F_(1,77)_ = 26.15, *p* < 0.05]. This shows that the IMA can significantly increase students' motivation for Tang poetry learning, and thus hypothesis 1 was accepted.

**Table 2:** Two groups of ANCOVA results based on results for the post-test on learning motivation.

**Group**	***N***	**Mean**	**S.D**.	**Adjusted mean**	**Std. error**	**F**
Experimental	40	65.35	2.99	65.37	0.44	26.15^**^
Control	40	62.18	2.59	62.16	0.44	

** p < 0.01.

Additionally, an ANCOVA was conducted to determine whether there is a significant difference between the post-test scores for intrinsic motivation and extrinsic motivation in the two groups. The post-test scores of intrinsic motivation between the experimental and control groups have reached a significant difference [F_(1,77_) = 28.16, *p* < 0.05], indicating that IMA can significantly increase students' intrinsic motivation. However, there was no significant difference between the two groups on the post-test scores for extrinsic motivation [F_(1,77)_ = 4.04, p > 0.05], as presented in Table 3. In other words, the IMA has not been able to increase the students' extrinsic motivation. Therefore, hypothesis 2 was partially accepted.

**Table 3:** Two groups of ANCOVA results based on the post-test for intrinsic and extrinsic motivation.

**Learning motivation**	**Group**	***N***	**Mean**	**S.D**.	**Adjusted mean**	**Std. error**	**F**
Intrinsic motivation	Experimental	40	35.48	1.65	35.47	0.26	28.16^**^
	Control	40	33.50	1.64	33.50	0.26	
Extrinsic motivation	Experimental	40	29.88	2.50	29.87	0.42	4.04
	Control	40	28.67	2.75	28.68	0.42	

** p < 0.01.

### 6.2. Learning comprehension

This section examines whether there is a significant difference in comprehension of Tang poetry between the experimental group using the IMA and the control group using traditional classroom instruction. An ANCOVA was performed on the post-test scores using the pre-test scores as covariant.

Tests for homogeneity of regression slopes and homogeneity of variance were performed on the comprehension scores of the two groups to meet the basic assumptions of analysis of covariance (ANCOVA). The assumption of homogeneity of regression slopes was not violated (F = 0.05, *p* = 0.82 > 0.05), indicating consistency between the covariant and dependent variables, and Levene's test for equality of error variances (*p* = 0.63 > 0.05) was not violated either. In addition, homogeneous regression slopes tests and homogeneity of variance tests were conducted for the multiple-choice section and the reading comprehension section, i.e., literal and inferential comprehension. The results showed that both dimensions did not violate the assumption of homogeneity of regression slopes with literal comprehension (F = 0.20, *p* = 0.66 > 0.05) and inferential comprehension (F = 0.61, *p* = 0.44 > 0.05), while the results of Levene's test of equality of error variances were not significant with literal comprehension (*p* = 0.92 > 0.05) and inferential comprehension (*p* = 0.24 > 0.05). Therefore, ANCOVA can be used to analyze both the literal and inferential comprehension dimensions. The results of the ANCOVA are summarized in Table 4.

**Table 4:** ANCOVA result for the comprehension post-test of the experimental and control groups.

**Group**	**N**	**Mean**	**S.D**.	**Adjusted mean**	**Std. error**	**F**
Experimental	40	33.28	5.11	33.42	0.76	14.93^**^
Control	40	29.40	5.02	29.25	0.76	

** p < 0.01.

The results indicate that, in general, the experimental and control groups showed significant differences in comprehension [F _(1,77)_ = 14.93, *p* < 0.05]. A higher mean score was achieved by the experimental group (33.28) than that by the control group (29.40). Therefore, hypothesis 3 is accepted. The result also manifested that the IMA is capable of promoting students' comprehension of Tang poetry.

An ANCOVA was also conducted on inferential comprehension and literal comprehension to determine whether there was a significant improvement in either. As illustrated in Table 5 both inferential comprehension and literal comprehension showed significant differences. On the inferential comprehension dimension, the mean scores for the experimental group was 19.38 and that of the control group was 16.50, with a significant increase [F_(1,77)_ = 12.12, *p* < 0.05]. Similar results were found for literal comprehension scores although the scores of literal comprehension by both groups were slightly lower than those in inferential comprehension dimension, with a mean of 13.90 for the experimental group and 12.90 for the control group, with a significant difference [F_(1,77)_ = 4.18, *p* < 0.05]. Based on the results of analysis on inferential and literal comprehension, the hypothesis 4 was accepted. The results of the analysis confirmed that students were able to improve both their inferential and literal comprehension of Tang poetry by using the IMA.

**Table 5:** Two groups of ANCOVA results based on the post-test for literal and inferential comprehension.

**Comprehension**	**Group**	**N**	**Mean**	**S.D**.	**Adjusted mean**	**Std. error**	**F**
Inferential comprehension	Experimental	40	19.38	4.11	19.42	0.60	12.12^**^
	Control	40	16.50	3.62	16.45	0.60	
Literal comprehension	Experimental	40	13.90	2.39	13.97	0.39	4.18^*^
	Control	40	12.90	2.79	12.83	0.39	

* p < 0.05;

** p < 0.01.

## 7. Discussion and conclusion

### 7.1. Discussion

In this study, an IMA was designed to investigate whether it has a positive effect on the students' motivation and comprehension when learning Tang poetry. In order to test the research hypothesis, a pretest-posttest control group experiment was conducted.

As for research hypothesis 1, the hypothesis aimed to compare motivation for learning Tang poetry in the experimental group using the IMA and the control group using traditional classroom teaching methods. The results of the study indicate a significant difference between the experimental and control groups, indicating that the IMA has the effect on improving students' motivation in learning Tang poetry. The hypothesis 2 is to explore whether there is a significant difference between intrinsic and extrinsic motivation among students in learning Tang poetry. Based on the results of the experiment, students who studied with the IMA demonstrated significantly higher intrinsic motivation than those using traditional classroom methods.

According to the CATLM, cognitive and affective processes are interdependent and interconnected (Moreno, 2006a). In interactive multimodal learning environments, motivational and affective elements are intertwined and integrated with each other, which can stimulate students' interest and enhance their intrinsic motivation for learning (Moreno, 2005). This is supported by Patall et al. (2008) and Jovanovic and Matejevic (2014) that the enhancement of students' intrinsic motivation is shown when students learn using interactive media, which then helps them to have a deeper comprehension of the learning content and results in better academic performance. Such findings are also in consistent with Wang et al.'s (2019) study. When learners are able to control their learning pace in interactive multimodal learning environments, it has a significant influence on their attitudes and motivations to learn and leads to the enhancement of their academic performance (Korat and Falk, 2019; Ahmed and Osman, 2020). Together with the improved results of students' comprehension on Tang poetry, it can be concluded that IMA learning is a facilitator on students' comprehension of Tang poetry, thus triggering their interest and intrinsic motivation for the course.

Regarding the comprehension of Tang poetry, it was found that students who used the IMA for Tang poetry had higher scores on the comprehension post-test compared to the control group using traditional classroom teaching methods. The results prove the effectiveness of using the IMA for learning Tang poetry. It was also found that students in the experimental group who used the IMA to learn Tang poetry performed better than the control group on both literal and inferential comprehension dimensions. Other studies also confirmed that students benefited a lot from an interactive multimodal learning environment where their reading comprehension and learning engagement were largely enhanced (Drigas et al., 2015; Kelley and Kinney, 2017; Christ et al., 2019; Bus et al., 2020). A number of studies focusing on interactive features indicate that interactive elements related to stories (e.g., hotspots closely integrated with the storyline) as well as those designed to enhance learning (e.g., fun quizzes) can enhance the learning process (Kao et al., 2016; Ross et al., 2016; Ghalebandi and Noorhidawati, 2019; Korat et al., 2022). Korat et al. (2022) found that ebooks with multimedia functions that focus on the storyline, including prompts for background knowledge and/or additional explanations, performed better than those without enhancements and received more preferences from users.

### 7.2. Conclusion

In summary, this study provides an effective method for enhancing students' reading comprehension and intrinsic motivation to learn Tang poetry, one of the most valuable cultural heritages of the Chinese classical literature, and this study provides new insight into the teaching and learning of Tang poetry.

Practically speaking, the use of the IMA for teaching Tang poetry is an attempt to introduce a new teaching method. By using this method, students can learn Tang poetry in an enjoyable and interactive environment, which can significantly enhance their motivation in learning. With the IMA applied, the comprehension of students on Tang poetry is improved. Students gained knowledge of Tang poetry, the background knowledge of poets and were guided to comprehend interpretation of Tang poetry in an interactive learning environment, with their intrinsitic motivation fostered. Moreover, the results may inspire language instructors or researchers in schools to actively have investigation on the content design of teaching Tang poetry, encourage them to introduce more technology-assisted teaching methods for building an interactive learning environment and combine technology and teaching in a reasonable manner to achieve good results. Additionally, this new learning model may be extended to other courses in Chinese.

The application of IMA to the study of Tang poetry has demonstrated a positive impact on primary school students' internal motivation and comprehension of this literary genre. As a result, this innovative pedagogical approach can also be used for the study of other ancient Chinese literary works such as song lyrics, Yuan opera, and the Analects of Confucius, which are also written in classical Chinese. These literary works are often challenging for students who are unfamiliar with the dense Chinese vocabulary and intricate phrasing, so the beneficial effects of IMA may similarly enhance the learning experience in these areas (Sung et al., 2022). For example, the Analects of Confucius, like Tang poetry, can be a complex text to understand for students who are unfamiliar with the classical Chinese style of expression (Sung et al., 2017). Meanwhile, the study used an IMA that followed the instructional design principles of CATLM, focusing on guided activities, learning activities, practice and feedback, and reflection. These design principles are transferable to other subjects and can be implemented by language teachers who wish to improve their IMA design and delivery using the ideas explored in this study.

All in all, from a theoretical standpoint, the results of this study validate the CATLM as well as CATLM-based instructional design principles and assumptions. Experimental data indicates that CATLM-based instructional design is effective in improving students' reading comprehension on Tang poetry and intrinstic motivation in learning. From a methodological standpoint, this study proves that the self-designed interactive multimodal application can be used as a facilitator on students' motivation in learning and is transferable to teaching other topics or subjects. From a pedagogical standpoint, the study illustrates that allowing learners to manage teaching materials presented in interactive multimodal environments is worth being considered further in teaching practice as a useful tool.

## 8. Limitation and recommendation

According to the current study, the IMA contributes to the improvement of students' motivation and comprehension of Tang poetry, but there are some limitations to this study. It was recommended that large-scale experiments be conducted in the future to better understand the effects of this approach since the study was conducted among only a small group of 80 third-grade students in an elementary school. Additionally, since this experiment was conducted in Xinzheng, Henan Province, the students may not be representative of students in other geographical areas due to differences in economic development and educational quality. Thus, the findings of this study need to be interpreted with caution if they are applied to other contexts. We hence call for more related studies on IMA in different contexts, with the consideration of local features such as cultural values and educational quality. Meanwhile, it is imperative to acknowledge that the implementation of the IMA could potentially cause a considerable amount of workload and psychological strain on teachers, as it may necessitate additional training and preparation in comparison to conventional teaching approaches. Finally, since the participants in this study were only involved third-grade students on Tang poetry learning, these results may not be easily generalized or transferable to other grades or teaching other subjects. Further research could explore the IMA's efficiency in other grades, for example, secondary, tertiary, or vocational education and other disciplines.

## Data availability statement

The raw data supporting the conclusions of this article will be made available by the authors, without undue reservation.

## Ethics statement

The studies involving human participants were reviewed and approved by Academic Ethics Committee of the School of Art and Design, Zhengzhou University of Industrial Technology. Written informed consent to participate in this study was provided by the participants' legal guardian/next of kin.

## Author contributions

CC and NJ contributed to the design and implementation of the research, the analysis of the results, and writing of the manuscript. All authors contributed to the article and approved the submitted version.
